# Fine Needle Biopsy Versus Core Needle Biopsy Combined With/Without Thyroglobulin or BRAF 600E Mutation Assessment for Detecting Cervical Nodal Metastasis of Papillary Thyroid Carcinoma

**DOI:** 10.3389/fendo.2021.663720

**Published:** 2021-04-12

**Authors:** Xiaojun Zhang, Xu Zhang, Wei Du, Liyuan Dai, Ruihua Luo, Qigen Fang, Hong Ge

**Affiliations:** ^1^ Department of Head Neck and Thyroid, Affiliated Cancer Hospital of Zhengzhou University, Henan Cancer Hospital, Zhengzhou, China; ^2^ Department of Radiation Oncology, Affiliated Cancer Hospital of Zhengzhou University, Henan Cancer Hospital, Zhengzhou, China

**Keywords:** papillary thyroid carcinoma, lymph node metastasis, FNAB-C, FNAB-Tg, BRAF V600E

## Abstract

**Objectives:**

To analyze the diagnostic benefit of fine needle aspiration biopsy cytology (FNAB-C) and core needle biopsy tissue (CNB-T) with the addition of thyroglobulin (Tg) in the washout of the needle or BRAF V600E mutation assessment in assessing cervical lymph node metastasis (LNM) in papillary thyroid carcinoma.

**Materials and Methods:**

A total of 186 lymph nodes were punctured by fine or core needle. The diagnostic performance of FNAB-C and CNB-T with Tg in the washout or BRAF V600E mutation assessment was compared.

**Results:**

The optimal cutoff value of FNAB-Tg was 1.0 ng/ml, with an AUC of 0.976. The sensitivity and specificity of FNAB-C in predicting cervical LNM were 97.4% and 71.4%, respectively, and the addition of FNAB-Tg could contribute to a sensitivity of 100% and a specificity of 95%, but the introduction of BRAF V600E mutation assessment was associated with a decreased sensitivity of 96.3% and a decreased specificity of 50.0%. The FNAB-Tg level showed a comparable distribution in malignant lymph nodes with different TgAb statuses, serum TSH levels, and serum Tg levels. The sensitivity and specificity of CNB-T in predicting cervical LNM were 98.9% and 100%, respectively. The addition of CNB-Tg did not alter the diagnostic ability, but the introduction of BRAF V600E mutation assessment obtained the best performance, with a sensitivity of 100% and specificity of 100%.

**Conclusion:**

The sensitivity and specificity of FNAB-C could be increased if combined with FNAB-Tg. CNB-T alone could provide satisfactory diagnostic reliability.

## Introduction

Papillary thyroid carcinoma (PTC) is the most common endocrine malignancy and accounts for more than 90% of all thyroid cancers (1). Unilateral or bilateral thyroidectomy combined with central neck dissection is the routine method of operation according to our guidelines (2). Lateral neck dissection is performed if cervical lymph node metastasis (LNM) is pathologically confirmed preoperatively or intraoperatively. Even if the operation is successful, a number of patients suffer from regional recurrence (3, 4); for these patients, it is important for the surgeon to accurately assess the lesion status preoperatively to avoid unnecessary surgery.

Ultrasound-guided fine needle aspiration biopsy cytology (FNAB-C) and core needle biopsy tissue (CNB-T) play an important role in evaluating cervical lymph node status in thyroid cancer and head and neck squamous cell carcinoma (SCC), respectively (5–21). However, the value of FNAB-C is greatly limited if the number of specimens is small or the specimens are obtained from cystic lymph nodes. Since it was first described by Pacini et al. (11), the measurement of thyroglobulin (Tg) in the washout of the needle (FNAB-Tg) has attracted increasing attention because of its higher reliability than FNAB-C (12–15). However, there is still great controversy regarding the best cutoff value of FNAB-Tg and whether serum thyroid stimulating hormone (TSH), thyroglobulin antibody (TgAb), serum Tg, and thyroid tissue interfere with the accuracy of FNAB-Tg (16, 17). Some researchers have reported that the clinical performance of FNAB-Tg was not affected by TgAb and TSH (10, 12), but others have asserted that the diagnostic value of FNAB-Tg could be greatly influenced by the presence of thyroid tissue, serum Tg, and TgAb (7, 14, 15, 18).

No researchers have yet analyzed the association between cervical LNM and CNB-T in thyroid cancer, so it remains unclear whether CNB-T is superior to FNAB-C in indicating LNM. In addition, the BRAF V600E mutation is the most common genetic alteration in thyroid cancer and occurs in nearly 80% of primary tumors and 50% of metastatic lymph nodes (22, 23). It therefore remains unknown whether the introduction of BRAF V600E mutation assessment could increase the diagnostic reliability of CNB-T and FNAB-C.

Thus, in the current study, we aimed to determine the answer to optimal value of Tg in the washout and compare the diagnostic performance between CNB-T and FNAB-C combined with/without Tg or BRAF 600E mutation assessment.

## Patients and Methods

### Ethics Statement

The Our Hospital Institutional Research Committee approved our study, and all participants provided written informed consent. All methods were performed in accordance with the relevant guidelines and regulations. All procedures performed in studies involving human participants were in accordance with the ethical standards of the institutional and/or national research committee and with the 1964 Declaration of Helsinki and its later amendments or comparable ethical standards.

### Patient Selection

From January 2018 to October 2020, medical records of patients with surgically treated primary or recurrent/metastatic PTC were retrospectively reviewed. Enrolled patients must meet the following criteria: the patient underwent preoperative fine or core needle biopsy for suspicious lymph nodes and the punctured lymph nodes had been surgically excised. Then, 306 patients who had lymph node biopsy for suspicious metastasis from PTC were selected. A total of 143 patients had negative CNB-T or FNAB-C results and did not receive surgical treatment for the lymph nodes, and 8 patients had positive CNB-T or FNAB-C results but did not receive surgical treatment for the lymph nodes. These 151 patients were excluded, therefore, a total of 155 patients were enrolled for analysis.

### Treatment Principles

Based on our guidelines for thyroid cancer (2), central neck dissection was routinely performed on patients treated for the first time, and lateral lymph node status was first assessed by ultrasound if there was suspicion of lateral LNM, FNAB-C or CNB-T combined with or without Tg in the washout or BRAF 600E mutation assessment.

For patients suspected of having regional recurrence, FNAB-C or CNB-T combined with or without Tg in the washout or BRAF 600E mutation assessment was performed for lateral neck lymph nodes, and FNAB-C or CNB-T combined with or without BRAF 600E mutation assessment was performed for central neck lymph nodes.

Cervical LNM was suggested if there were the following features, for ultrasound: absence of an echogenic hilum, round shape, microcalcification, peripheral blood flow on color Doppler images, or cystic changes (7–18); for CT: area with clear evidence of nonfat, low-density, or liquid components; largest diameter >15 mm at level II and >10 mm at other levels; and ratio of the longest to smallest diameter ≤2.

The FNAB-C and CNB-T results were both classified into three groups: inadequate or nondiagnosis, negative, or positive for metastatic PTC. Immunohistochemistry was performed if there was a need for accurate diagnosis.

For patients treated for the first time, lateral neck dissection was performed if there was a positive FNAB-C or CNB-T result regardless of the value of Tg in the washout or BRAF 600E status; if there was a negative or nondiagnosis FNAB-C or CNB-T result but high Tg in the washout or BRAF 600E mutation assessment, frozen sectioning of the suspicious lymph nodes was also performed.

For patients suspected of having regional recurrence, an operation was performed if there was a positive FNAB-C or CNB-T result regardless of the Tg value in the washout or BRAF 600E status; if there was a negative or nondiagnosis FNAB-C or CNB-T result but high Tg in the washout or BRAF 600E mutation assessment, different procedures, including repeated aspiration, close monitoring, direct surgery, etc., were selected accordingly.

### Important Variable Definition

The normal ranges for serum TSH, Tg, and TgAb were from 0.27 mIU/L to 4.2 mIU/L, from 3.5 ng/ml to 77 ng/ml, and from 0 to 115 IU/ml, respectively. TgAb(+) was indicated if its value was higher than 115 IU/ml.

### Fine and Core Needle Aspiration Technique and BRAF 600E Mutation Assessment

Fine needle aspiration biopsy was performed using a 22-gauge, and core needle biopsy was performed using an 18-gauge needle. Each aspiration was repeated at least three times to allow cytological or tissue examination and Tg in the washout determination or BRAF 600E mutation assessment. Immediately after aspiration, the needle and syringe were washed with 1 ml of normal saline and then sent to the laboratory if the Tg assay was required. A sufficient sample with an adequate number of tumor cells was used for real-time PCR analysis if BRAF 600E mutation assessment was required.

### Statistical Analysis

The final outcome was confirmed by postoperative pathology, and the diagnostic abilities of FNAB-C and CNB-T were assessed with respect to their sensitivity, specificity, positive predictive value (PPV), and negative predictive value (NPV). The Mann-Whitney U test was used to compare the FNAB-Tg values between different groups. All statistical analyses were performed with SPSS 20.0, and p<0.05 was considered to be significant.

## Results

### Characteristics of the Patients and Lymph Nodes

Finally, 155 patients (50 male and 105 female) were enrolled, with a mean age of 43.7 (range: 19-78) years. Ninety-five (61.3%) patients had received no prior treatment, 49 (31.6%) patients had previously undergone total thyroidectomy, and 11 (7.1%) patients had previously received unilateral thyroid lobectomy (**Table 1**). A total of 186 solid lymph nodes were punctured and surgically treated; among them, 88 (47.3%) lymph nodes underwent fine needle aspiration and 98 (52.7%) lymph nodes underwent core needle aspiration. Fifteen (8.1%) lymph nodes were located at level 6, and 171 (91.9%) lymph nodes were located at the lateral neck (levels 2-5). Postoperative pathology confirmed metastatic disease in 174 (92.6%) lymph nodes. BRAF 600E mutation assessment was performed in 88 lymph nodes, and a mutation was identified 41 (46.6%, 41/88) patients. Tg in the washout was evaluated in 98 lymph nodes, and its value was less than 1 ng/ml in 13 (13.3%, 10/98) patients and greater than 1 ng/ml in 85 (86.7%, 88/98) patients (**Table 2**).

**Table 1:** Information of the enrolled 155 patients.

Variables	Patients (%)
Age	
<55	115 (74.2%)
≥55	40 (25.8%)
Sex	
Male	50 (32.3%)
Female	105 (67.7%)
Prior treatment	
None	95 (61.3%)
Total thyroidectomy	49 (31.6%)
Unilateral thyroid lobectomy	11 (7.1%)

**Table 2:** Information of the 186 punctured lymph nodes.

Variables	Number (%)
Puncture type	
Fine needle	88 (47.3%)
Core needle	98 (52.7%)
Location	
Level 2-5	171 (91.9%)
Level 6	15 (8.1%)
Postoperative pathology	
Malignant	174 (93.5%)
Benign	12 (6.5%)
BRAF 600E mutation assessment	
Positive	41 (46.6%, 41/88)
Negative	47 (53.4%, 47/88)
Tg in the wash-out	
<1ng/ml	13 (13.3%, 10/98)
≥1ng/ml	85 (86.7%, 88/98)

### ROC Curve of the Optimal Cutoff Value of FNAB-Tg

**Figure 1:**
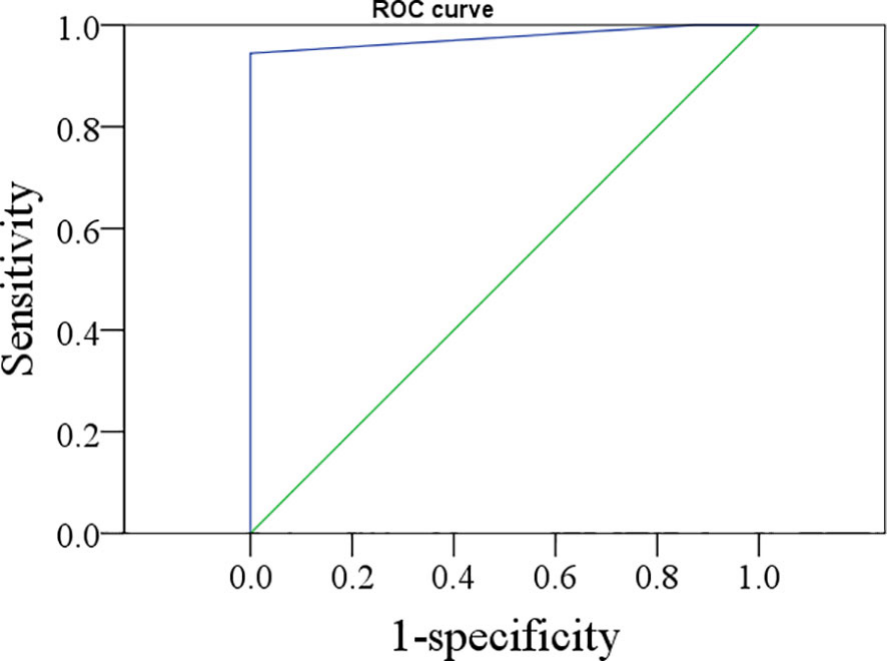
The ROC curve of thyroglobulin in the washout of the needle for indicating lymph node metastasis.

According to the ROC analysis (**Figure 1**), the optimal cutoff value of FNAB-Tg was 1.0 ng/ml, with an AUC of 0.976, and its sensitivity and specificity in predicting cervical LNM were 94.4% and 100%, respectively.

### Diagnostic Performance Between FNAB-C and CNB-T

In 78 lymph nodes with positive FNAB-C results, 76 lymph nodes were confirmed to be metastatic by positive pathology; 7 lymph nodes had negative results, and 2 lymph nodes were confirmed to be metastatic by positive pathology. The sensitivity and specificity of FNAB-C in predicting cervical LNM were 97.4% and 71.4%, respectively (**Table 3**).

**Table 3:** Diagnostic performance of between FNAB-C and CNB-T.

	Postoperative pathology	
	Malignant	Benign
FNAB-C		
Positive	76 (97.4%)	2 (28.6%)
Negative	2 (2.6%)	5 (71.4%)
CNB-T		
Positive	91 (98.9%)	0
Negative	1 (1.1%)	6 (100%)

In 91 lymph nodes with positive CNB-T results, all lymph nodes were confirmed to be metastatic by positive pathology; in 7 lymph nodes had negative results, and 1 lymph node was confirmed to be metastatic by positive pathology. The sensitivity and specificity of CNB-T in predicting cervical LNM were 98.9% and 100%, respectively (**Table 3**).

### Effect of BRAF 600E Mutation Assessment on the Diagnostic Performance of FNAB-C and CNB-T

In 32 lymph nodes, both FNAB-C and BRAF 600E mutation assessments were performed; in 28 lymph nodes with positive FNAB-C results or BRAF 600E mutation assessments, 27 lymph nodes were found to be metastatic. The sensitivity and specificity of FNAB-C combined with BRAF 600E mutation assessment in predicting cervical LNM were 96.4% and 50%, respectively (**Table 4**).

**Table 4:** Diagnostic performance in FNAB-C and CNB-T combined with or without Tg in the wash-out or BRAF 600E mutation assessment.

	Sensitivity	Specificity	PPV	NPV
FNAB-C	97.4%	71.4%	97.4%	71.4%
FNAB-C and FNAB-Tg	100%	95%	98%	100%
FNAB-C and BRAF 600E mutation	96.4%	50.0%	96.4%	50.0%
CNB-T	98.9%	100%	100%	85.7%
CNB-T and CNB-Tg	97.6%	100%	100%	66.7%
CNB-T and BRAF 600E mutation	100%	100%	100%	100%

In 56 lymph nodes, both CNB-T and BRAF 600E mutation assessments were performed; in 52 lymph nodes with positive CNB-T results or BRAF 600E mutation assessments, all lymph nodes were found to be metastatic. The sensitivity and specificity of CNB-T combined with BRAF 600E mutation assessment in predicting cervical LNM were 100% and 100%, respectively (**Table 4**).

### Effect of Tg on the Diagnostic Performance of FNAB-C and CNB-T in the Washout

In 55 lymph nodes, both FNAB-Tg and FNAB-C were performed, and all 49 lymph nodes with positive FNAB-C results or high FNAB-Tg (≥1 ng/ml) were confirmed to be metastatic postoperatively. The sensitivity and specificity of FNAB-C combined with FNAB-Tg in predicting cervical LNM were 100% and 95%, respectively (**Table 4**).

In 43 lymph nodes, both CNB-Tg and CNB-T were performed, and all 40 lymph nodes with positive CNB-T results or high CNB-Tg (≥1 ng/ml) were confirmed to be metastatic postoperatively. The sensitivity and specificity of CNB-T combined with CNB-Tg in predicting cervical LNM were 97.6% and 100%, respectively (**Table 4**).

### Association Between TgAb, Serum TSH, Serum Tg, Thyroid Tissue and FNAB-Tg

In 40 positive lymph nodes from TgAb(+) patients, the mean FNAB-Tg value was 228.1 ± 211.8 ng/ml, and in 58 positive lymph nodes from TgAb(-) patients, the mean FNAB-Tg value was 284.1 ± 211.8 ng/ml; the difference was not significant (p=0.188). (**Table 5**)

**Table 5:** Distribution of FNAB-Tg in positive lymph nodes with different characteristics.

Characteristic	FNAB-Tg (ng/ml)	p
TgAb		
Positive	228.1 ± 211.8	
Negative	284.1 ± 211.8	0.188
Serum TSH		
High	224.1 ± 184.9	
Normal	263.3 ± 214.1	
Low	293.3 ± 211.6	0.413
Serum Tg		
High	282.0 ± 210.0	
Normal	252.7 ± 204.9	
Low	260.0 ± 207.6	0.846
Thyroid tissue		
Yes	264.1 ± 207.2	
No	214.9 ± 194.0	0.331

In 36 positive lymph nodes from low TSH patients, the mean FNAB-Tg value was 293.3 ± 211.6 ng/ml, in 35 lymph nodes from normal TSH patients, the mean FNAB-Tg value was 263.3 ± 214.1 ng/ml, and in 27 lymph nodes from high TSH patients, the mean FNAB-Tg value was 224.1 ± 184.9 ng/ml. However, this difference was not significant (p=0.413) (**Table 5**).

In 33 positive lymph nodes from low serum Tg patients, the mean FNAB-Tg value was 260.0 ± 207.6 ng/ml, in 35 lymph nodes from normal serum Tg patients, the mean FNAB-Tg value was 252.7 ± 204.9 ng/ml, and in 30 lymph nodes from high serum Tg patients, the mean FNAB-Tg value was 282.0 ± 210.0 ng/ml. However, this difference was not significant (p=0.846) (**Table 5**).

In 23 positive lymph nodes from patients without thyroid tissue presence, the mean FNAB-Tg value was 214.9 ± 194.0 ng/ml, and in 75 positive lymph nodes from patients with thyroid tissue presence, the mean FNAB-Tg value was 264.1 ± 207.2 ng/ml. However, this difference was not significant (p=0.331) (**Table 5**).

### Association Between TgAb, Serum TSH, Serum Tg, Thyroid Tissue and the Diagnostic Performance of FNAB-Tg

As described in **Table 6**, both the sensitivity and NPV were 100% in positive lymph nodes with different characteristics, and both the specificity and PPV had a variation range of less than 5.0%.

**Table 6:** Association between TgAb, serum TSH, serum Tg, thyroid tissue and the diagnostic performance of FNAB-C and FNAB-Tg in positive lymph nodes.

Characteristics	Diagnostic performance of FNAB-C and FNAB-Tg			
	Sensitivity	Specificity	PPV	NPV
TgAb				
Positive	100%	98.4%	100%	100%
Negative	100%	94.1%	96.9%	100%
Serum TSH				
High	100%	92.3%	100%	100%
Normal	100%	94.7%	96.7%	100%
Low	100%	97.8%	98.3%	100%
Serum Tg				
High	100%	93.7%	98.1%	100%
Normal	100%	95.2%	100%	100%
Low	100%	96.9%	97.2%	100%
Thyroid tissue				
Yes	100%	96.6%	99.0%	100%
No	100%	93.5%	97.2%	100%

## Discussion

The most important finding in the current study was that the optimal cutoff value of FNAB-Tg was 1.0 ng/ml, and its addition increased the sensitivity and specificity of FNAB-C. The diagnostic performance was not affected by TgAb, serum TSH, serum Tg, or thyroid tissue. CNB-T alone could provide satisfactory diagnostic reliability, and the additional benefit was greater with BRAF 600E mutation assessment than with FNAB-Tg. In all groups, CNB-T combined with BRAF 600E mutation assessment had the best diagnostic ability.

The optimal cutoff of FNAB-Tg value had been studied previously and reported to be in the range of 0.9 ng/ml to 50 ng/ml (17). A meta-analysis by Grani et al. (17) indicated a pooled sensitivity of 94.8% and a specificity of 91.2% with a cutoff of 0.9-1.1 ng/ml, a pooled sensitivity of 87.7% and a specificity of 94.2% with a cutoff of 10 ng/ml, and a pooled sensitivity of 97.3% and specificity of 94.9% with a cutoff of serum Tg in predicting cervical NLM. Uruno et al. (5) analyzed 129 fine needle punctured lymph nodes, of which 105 lymph nodes were positive by FNAB-Tg (range 6.2-8000 ng/ml) and 24 lymph nodes were negative (range 0.6-88.8 ng/ml). FNAB-Tg and FNAB-C can compensate for the loss of the other based on the assumption that the lymph node is positive if the FNAB-Tg value is higher than the serum Tg level, taking blood contamination into consideration. However, in practice, the sample for FNAB-Tg is diluted with 1 ml normal saline, with a typical dilution between 50 and 200. In addition, Borel et al. (24) reported that the maximum blood contamination of the needle washout fluid in theory was approximately 20% and contamination was less than 5% in experiments evaluating albumin concentration in FNAB washout fluid; therefore, even if blood contamination did occur, the FNAB-Tg value was still lower in a negative lymph node than in a positive lymph node. This finding was supported by our ROC results. Pathology results are typically the gold standard for decision making in thyroid cancer; therefore, the main contribution of FNAB-Tg assessment is in determining the best balance between necessary and unnecessary surgery in metastatic patients with a negative or nondiagnostic pathological result. Konca Degertekin et al. (12) analyzed the diagnostic accuracy of FNAB-C and FNAB-Tg in 51 lymph nodes and found that FNAB-Tg≥1 ng/ml was associated with similar accuracy and even a higher specificity and PPV compared to FNAB-C alone, and the authors concluded that the best cutoff value was 1.0 ng/ml. Moon et al. (25) reported that the median FNAB-Tg was 521.2 ng/ml in malignant lymph nodes, and the optimal cutoff value of FNAB-Tg was 1.0 ng/ml, with a sensitivity of 93.2% and a specificity of 95.9%. Combining FNAB-Tg and FNAB-C showed superior diagnostic power compared with the diagnostic strategy of using either FNAB-C or FNAB-Tg alone. The finding was consistent with our own results: a cutoff value of 1.0 ng/ml is related to high sensitivity and specificity.

However, factors that could affect FNAB-Tg need to be analyzed further. Li et al. (18) described 6 cases with very elevated FNAB-Tg levels that were confirmed to be normal thyroid tissue, in which 3 cases were labeled as metastatic lymph nodes at level 6. It has been accepted that residual thyroid tissue might be mistaken for suspicious lymph nodes in patients with a history of thyroid surgery, and this viewpoint was confirmed by Jeon et al. (7) and Tang et al. (15): some cases with high FNAB-Tg levels were ultimately explained by remnant thyroid tissue. Therefore, caution must be taken when a negative FNAB-C result but a high FNAB-Tg level is observed in samples collected from the central neck. However, the situation in lateral cervical lymph nodes is more difficult to discern. Usually, the needle path does not pass through the thyroid region during puncture for levels 2-5, and our results also confirmed that there was no thyroid tissue to be considered for a negative FNAB-C but positive FNAB-Tg.

The interference effect of TgAb is most frequently evaluated in the literature but remains unclear. Jo et al. (14) demonstrated that the FNAB-Tg level was significantly higher in TgAb- patients than in TgAb+ patients, and its sensitivity and PPV were increased by >10% in TgAb- patients compared to TgAb+ patients when using the same cutoff value. However, Konca Degertekin et al. (12) reported that TgAb+ and TgAb- patients had comparable FNAB-Tg levels in malignant lymph nodes, and the diagnostic performance of FNAB-Tg was independent of TgAb status. Similar findings were also published by Boi et al. (10) and Duval et al. (26). There were at least two explanations for this finding: on the one hand, TgAb was negative in FNAB from patients with positive serum TgAb (27), and on the other hand, an elevated FNAB-Tg level can easily override the effect of TgAb caused by blood contamination.

The role of TSH and serum Tg has only been analyzed occasionally. One study commented that TSH increased the possibility of false-negative FNAB-Tg in malignant cases (7), but there was no strong evidence to support this viewpoint. In our opinion, blood contamination is a direct or indirect cause that could explain the effect of TSH and serum Tg. In the current study, FNAB-Tg was at the same level in patients with positive lymph nodes who had different TSH and serum Tg levels, and the diagnostic performance of FNAB-C had little relationship with TSH and serum Tg. This finding might be partially due to the abilities of our experienced thyroidologists and cytopathologists.

BRAF 600E mutation was the most common genetic change in PTC patients with geographical differences and occurred in most of the primary tumors and metastatic lymph nodes. Chen et al. (23) reported that 78% of positive lymph nodes had BRAF 600E gene changes, but the mutation was not associated with the number, extranodal extension, or stage of the positive lymph nodes. A slightly lower rate of 47.1% was reported by Kurtulmus et al. (22), but the authors found that the presence of the BRAF 600E mutation in lymph nodes was not affected by mutation in the primary tumor. This finding introduced the possibility of BRAF 600E mutation assessment in predicting cervical LNM. Li et al. (28) might be the only author to evaluate the utilization of BRAF 600E mutation assessment in diagnosing cervical LNM; in their study, 27 positive lymph nodes had a negative FNAB-C result, but 17 of the 27 cases were positive for BRAF 600E mutation. The authors concluded that BRAF 600E mutation assessment could provide diagnostic support in PTC patients, but the benefit extent was small. In our study, it was also noted that compared to FNAB-C alone, the diagnostic performance of FNAB-C combined with BRAF 600E mutation assessment was reduced. One possible explanation is that the samples collected from fine needle puncture ready for BRAF 600E mutation assessment were very limited, increasing the misdiagnosis rate.

Core needle biopsy is another method for disease diagnosis. Novoa et al. (21) confirmed its reliability based on a systemic analysis revealing that CNB provided a correct specific diagnosis in 87% of cases without major complications; however, no other authors have tried to evaluate the feasibility of CNB in predicting cervical LNM in PTC. We were the first to report the high sensitivity and specificity of CNB alone, which could be explained by the fact that only clinically suspicious lymph nodes were punctured, and all the procedures were performed by experienced clinicians. It is interesting to note that the diagnostic ability of CNB-T is decreased when combined with CNB-Tg, and one possible explanation is that blood contamination is very frequent during CNB. However, the relationship between CNB-T and BRAF 600E mutation assessment can allow for the best diagnostic performance and prevent all unnecessary surgeries.

The current study has some limitations that must be acknowledged. First, our sample size was relatively small, which might have decreased our statistical power. Second, there is inherent bias in all retrospective studies. Third, we need to stay awake that the practicality of core biopsy on suspicious lymph nodes is limited in selective patients, especially the pediatric population, because core biopsy is more invasive than an fine needle aspiration.

In summary, the sensitivity and specificity of FNAB-C could be increased when combined with FNAB-Tg, with an optimal cutoff value of 1.0 ng/ml. CNB-T alone can provide satisfactory diagnostic reliability, and the additional benefit is greater with BRAF 600E mutation assessment than with FNAB-Tg.

## Data Availability Statement 

The original contributions presented in the study are included in the article. Further inquiries can be directed to the corresponding authors.

## Ethics Statement

The studies involving human participants were reviewed and approved by The Our Hospital Institutional Research Committee, and all participants provided written informed consent. The patients/participants provided their written informed consent to participate in this study.

## Author Contributions

All the authors made the contribution in study design, manuscript writing, studies selecting, data analysis, study quality evaluating, and manuscript revising. All authors contributed to the article and approved the submitted version.

## Conflict of Interest

The authors declare that the research was conducted in the absence of any commercial or financial relationships that could be construed as a potential conflict of interest.
